# Invaders in hot water: a simple decontamination method to prevent the accidental spread of aquatic invasive non-native species

**DOI:** 10.1007/s10530-015-0875-6

**Published:** 2015-03-26

**Authors:** Lucy G. Anderson, Alison M. Dunn, Paula J. Rosewarne, Paul D. Stebbing

**Affiliations:** 1 School of Biology, University of Leeds, Leeds, LS2 9JT UK; 2 Centre for Environment, Fisheries and Aquaculture Science (Cefas), Barrack Road, The Nothe, Weymouth, Dorset, DT4 8UB UK

**Keywords:** Angling equipment, Biosecurity, Invasive species management, Watersports equipment

## Abstract

Watersports equipment can act as a vector for the introduction and spread of invasive non native species (INNS) in freshwater environments. To support advice given to recreational water users under the UK Government’s *Check Clean Dry* biosecurity campaign and ensure its effectiveness at killing a range of aquatic INNS, we conducted a survival experiment on seven INNS which pose a high risk to UK freshwaters. The efficacy of exposure to hot water (45 °C, 15 min) was tested as a method by which waters users could ‘clean’ their equipment and was compared to drying and a control group (no treatment). Hot water had caused 99 % mortality across all species 1 h after treatment and was more effective than drying at all time points (1 h: χ^2^ = 117.24, *p* < 0.001; 1 day χ^2^ = 95.68, *p* < 0.001; 8 days χ^2^ = 12.16, *p* < 0.001 and 16 days χ^2^ = 7.58, *p* < 0.001). Drying caused significantly higher mortality than the control (no action) from day 4 (χ^2^ = 8.49, *p* < 0.01) onwards. In the absence of hot water or drying, 6/7 of these species survived for 16 days, highlighting the importance of good biosecurity practice to reduce the risk of accidental spread. In an additional experiment the minimum lethal temperature and exposure time in hot water to cause 100 % mortality in American signal crayfish (*Pacifastacus leniusculus*), was determined to be 5 min at 40 °C. Hot water provides a simple, rapid and effective method to clean equipment. We recommend that it is advocated in future biosecurity awareness campaigns.

**Electronic supplementary material:**

The online version of this article (doi:10.1007/s10530-015-0875-6) contains supplementary material, which is available to authorized users.

## Introduction

Invasive non native species (INNS) can have profound impacts on the marine, terrestrial and freshwater ecosystems they invade by replacing native species, altering community structure and introducing novel diseases (Mack et al. 2000). Freshwater systems are particularly vulnerable to the introduction of INNS due to their exposure to multiple transport pathways along which new species can be either accidentally or intentionally introduced. Moreover, the ecological resilience of freshwater systems is already reduced by pollution, agricultural run-off and altered hydrology (Strayer 2010), increasing the likelihood that non-native species will successfully invade (Dudgeon et al. 2006).

Fishing, boating and leisure activities are collectively responsible for almost 40 % of aquatic species introductions into Europe (Gallardo and Aldridge 2013). These pathways commonly include the release of boat ballast water and the stocking and subsequent escape of non-native fish or crustaceans introduced for aquaculture or sport. However, they also include the accidental transfer of invasive plants and invertebrate species “hitchhiking” on personal equipment such as angling nets, bait buckets, wet suits and waders used during recreational activities (Ludwig and Leitch 1996; Buchan and Padilla 1999; Johnson et al. 2001; Gates et al. 2008; Stebbing et al. 2011; Stasko et al. 2012; Bacela-Spychalska et al. 2013). Such accidental transfer is thought to have been responsible for new introductions, as well as facilitating the secondary spread of species once introduced (Johnson et al. 2001; Bothwell et al. 2009; Kilian et al. 2012).

Freshwater ecosystems in the UK contain seven of the UK Environment Agency’s 10 ‘most wanted’ INNS (Environment Agency 2011) and are thought to be threatened by a further 11 (Gallardo and Aldridge 2013). Many of these aquatic invasive species can survive for several days in damp environments. For example, zebra mussels can survive outside water for at least 5 days (Ricciardi et al. 1995) and killer shrimp (*Dikerogammarus villosus*) for at least 15 days (Fielding 2011). As 64 % of anglers visit more than one catchment within a fortnight (Anderson et al. 2014), it is likely that many aquatic INNS could survive the journey from an invaded catchment to an uninvaded catchment on damp equipment if effective biosecurity measures are not in place.

Once established, the eradication of these species is virtually impossible (Mack et al. 2000; Kolar and Lodge 2001; Briski et al. 2012) and control measures costly (Oreska and Aldridge 2010). Preventing their initial introduction and spread through effective biosecurity is therefore considered a far more effective management strategy (Vander Zanden et al. 2010; Caplat and Coutts 2011; Briski et al. 2012). The *Check Clean Dry* campaign was launched in the UK by the Government’s Department of Environment, Food and Rural Affairs (Defra) in 2010. The objective of the campaign is to raise awareness of good biosecurity practices among recreational water users to prevent the introduction and spread of aquatic INNS. The campaign provides broad guidance for best-practice:

**Check** your equipment and clothing for live organisms—particularly in areas that are damp or hard to inspect. **Clean** and wash all equipment thoroughly. If you do come across any organisms, leave them at the water body where you found them. **Dry** all equipment and clothing—some species can survive for many days in damp conditions. Make sure you don’t transfer water elsewhere.” (Defra 2013)

Specific advice about the most effective method by which to clean equipment is required.

Thermal control is considered to be one of the most efficient, environmentally sound and cost effective methods by which to prevent the accidental spread of aquatic INNS (O’Neill and MacNeill 1991; Beyer et al. 2010; Stebbing et al. 2011; Perepelizin and Boltovskoy 2011). Preliminary research conducted by the Centre for Environment, Fisheries and Aquaculture Science (Cefas) indicated that submersion in hot water at 45 °C was sufficient to cause 100 % mortality in *D. villosus* within 15 min (Stebbing et al. 2011). This advice has since been incorporated within local biosecurity awareness programmes (e.g. (Broads Authority 2013).

Hot water at this temperature meets the essential criteria for an effective cleaning treatment: it is accessible, economical, requires no specific training or protective equipment to use and has no impact on the environment when disposed (potentially in large volumes) (Kilroy et al. 2006). However, the recommended cleaning treatment needs to be effective at killing a wide range of aquatic INNS in addition to *D. villosus* as it is unrealistic to expect water users to use multiple treatments for different species, or to know which invasive species are present in different waterways.

Previous studies indicate that hot water can also cause 100 % mortality in zebra mussels (*D. polymorpha*), quagga mussels (*Dreissena rostriformis bugensis*) and the planktonic lifestage of spiny water fleas (*Bythotrephes longimanus*) (Beyer et al. 2010) as well as the invasive diatom didymo (*Didymo germinata*) (Kilroy et al. 2006) suggesting potential efficacy of this treatment across a range of taxonomic groups. Whether the 45 °C/15 min protocol is effective across multiple INNS, including plants, remains to be tested however.

The study had three aims: (1) to test whether the cleaning and drying components of the *Check Clean Dry* protocol were effective at killing a range of aquatic INNS should they become tangled in anglers keep nets; (2) to evaluate whether hot water at 45 °C is an effective method for killing a range of high impact aquatic INNS; and (3) to conduct a pilot experiment to test whether hot water could be a feasible biosecurity treatment for larger INNS such as American signal crayfish.

## Materials and methods

Survival experiments were conducted during October/November 2013 and February/March 2014 to evaluate the effectiveness of drying and hot water as treatments for decontaminating angling nets. Seven aquatic INNS representing a range of taxa and all currently present in the UK were used: zebra mussels (*Dreissena polymorpha*), killer shrimp (*D. villosus*), bloody-red mysid (*Hemimysis anomala*), floating pennywort (*Hydrocotyle ranunculoides*), curly water-thyme (*Lagarosiphon major*), New Zealand Pigmyweed (*Crassula helmsii*), and parrot’s feather (*Myriophyllum aquaticum*). Species were selected due to their classification as high impact invaders by the UK Technical Advisory Group for the EU Water Framework Directive.

A second experiment to test the effect of hot water temperature and duration of exposure on the survival of signal crayfish (*Pacifastacus leniusculus*) was conducted during March 2014. Adult crayfish were used as a proxy for juvenile crayfish (which may be difficult for anglers to detect) because juveniles were not accessible at the time of year when the experiment was undertaken. It was also reasoned that a treatment that is effective in killing adults is likely to also be effective for juveniles due limited ontogenetic changes in body morphology between juvenile and adult crayfish life stages (Holdich 2001).

The animals and plants were collected from various sites across the UK using hand searching (zebra mussels, killer shrimp, bloody-red mysid, signal crayfish) or from UK retailers of aquatic pond plants where it was unfeasible to collect wild specimens (*Lagarosiphon major*, parrot’s feather, New Zealand pigmyweed, curly water thyme). Once collected, plants/animals were stored in separate tanks of dechlorinated, aerated tap water at constant temperature (14 ± 1 °C, light: dark cycle 12: 12 h) for at least 48 h before the experiment to enable acclimation to laboratory conditions and recovery from collection or transport-induced stress. The temperature conditions were chosen to reflect the conditions in a garage or shed, the conditions in which most anglers store their equipment (Anderson et al. 2014).

### *Check Clean Dry* experiment

At the start of the experiment, plants were removed from the tank and cut into fragments of approximately 60 mm to simulate a fragment of plant that may become broken off and tangled up in an angling net. As the plant species were all vegetative reproducers, care was taken to include the reproductive part of the plant in each fragment. A *FluorPen* (FP 100, Photon Systems Instruments, Czech Republic) was used to determine the equivalent variable fluorescence: maximal fluorescence (F_V_:F_M_) ratio in the aquatic plants. This ratio is commonly used as an index of plant stress (Willits and Peet 2001). Only those with scores of at least 0.7 were deemed healthy and included in the experiment (Dan et al. 2000).

Zebra mussels, killer shrimp and bloody-red mysid were randomly selected from the tank to prevent bias towards particular sizes. Only those swimming normally (killer shrimp, bloody-red mysid) or siphoning water and responding to stimuli (zebra mussels) were used in the experiment (Beyer et al. 2010). Zebra mussels ranged in total length from 8.0 to 22.0 mm (median 16.0 mm), killer shrimp ranged from 8.7 to 20.9 mm (median 11.2 mm) and bloody-red mysid ranged from 10.5 to 13.8 mm (median 12.5 mm). There was no significant difference in the sizes of zebra mussels (Kruskal–Wallis, H = 2.1, *df* = 3, *p* = 0.55), killer shrimp (H = 3.17, *df* = 3, *p* = 0.36) or bloody-red mysid (H = 7.39, *df* = 3, *p* = 0.06) assigned to different treatments.

To mimic the conditions of an angler’s keep net, each animal or plant fragment (n = 240 per species) was placed in a bag (50 mm × 50 mm) constructed from the mesh (2 mm spacing) of a typical polyester coarse angling keep net (Keepnets Direct, UK). The bags were sealed with staples and submerged in dechlorinated tap water at 14 ± 1 °C for 1 h to simulate an angling trip. Once damp, the nets were subjected to one of three treatments: (1) hot water (45 °C); (2) hot water (45 °C) and drying, and (3) drying only; or a no-treatment control (see Table 1). For the hot water treatments, a 15 min exposure period was selected as this duration has been previously shown to be effective at causing 100 % mortality in killer shrimp (Stebbing et al. 2011) and because this is the maximum period of time that a treatment could realistically be applied in the field. For the drying treatments, net bags were laid on plastic trays in a temperature controlled room (14 ± 1 °C, light: dark cycle 12: 12 h, gently circulating air 1.23 m/s). In the control, net bags were placed in thin, transparent unsealed plastic bags to hinder drying and stored in the same way as the drying treatments. The relative rates at which the net bags dried in each treatment are supplied as supplementary material.

**Table 1:** Summary of experimental set up

Treatment	Description	Number of individuals checked at each time point					
		1 h	1 day	2 days	4 days	8 days	16 days
Hot water only	60× individual mesh nets submerged in a waterbath at 45 °C for 15 min in a randomly assigned order. Immediately afterwards, nets put inside individual (unsealed) plastic bags and stored on a tray in climate controlled room at 14 ± 1 °C	10	10	10	10	10	10
Hot water and drying	60 individual mesh nets submerged in water bath at 45 °C for 15 min in a randomly assigned order. Immediately afterwards, nets laid out on tray in climate controlled room at 14 ± 1 °C	10	10	10	10	10	10
Drying only	60 mesh nets laid out on trays in climate controlled room at 14 ± 1 °C	10	10	10	10	10	10
Control	60 mesh nets put inside individual (unsealed) plastic bags and stored on a tray in climate controlled room at 14 ± 1 °C	10	10	10	10	10	10

The description outlines the treatment that each polyester net (containing an individual animal or plant fragment, n = 240 per species) was exposed to after having been submerged in dechlorinated water at an ambient temperature for 1 h to simulate the minimum length of an angling trip

Animals/plants were observed and recorded as alive/dead at six time points after the initial treatment: 1 h, 1, 2, 4, 8, and 16 days. Our previous research indicated that 86 % of anglers use their equipment at least once a fortnight (Anderson et al. 2014) so the time units were chosen to represent time intervals during which angling equipment might be stored for between uses. Because the plants and animals had to be handled and/or exposed to water to test for survival, separate batches of 10 animals were tested at each time point. Having been tested, individuals were not returned to the experiment.

### Testing survival

Zebra mussels were assumed dead if their shells gaped and they did not respond to stimuli either immediately after the experiment or after 1 h recovery in a container of dechlorinated water at 14 ± 1 °C (Ricciardi et al. 1995; Beyer et al. 2010; Comeau et al. 2011). Killer shrimp and bloody-red mysid were considered dead if they were discoloured (or had begun to decompose) and neither responded to stimuli nor swam after being put in a container of dechlorinated water for 1 h. For the plants, a FluorPen was used at the end of the experiments to measure the variable to maximal fluorescence of leaves (F_v_:F_m_). This measurement is widely used as an indication of plant stress (Willits and Peet 2001), and plants with F_v_:F_m_ values of 0.3 or below were considered to be dead (Dan et al. 2000).

### Crayfish experiment

At the beginning of the experiment a single animal was removed from the holding tank, sexed, measured from the tip of the rostrum to the end of the cephalothorax (mm) and placed into a water bath at 30, 40, 50 or 60 °C (± 1 °C) for either 5, 1 min or 5 s for one of the temperatures (five animals per treatment × nine treatments). Where all individuals survived a treatment after 5 min, or 1 min, the treatment was not repeated for the shorter time period(s). Once the animal had been submerged for the required duration, it was removed and placed into dechlorinated water at 14 ± 1 °C for a recovery period of 30 min. Behavioural observations to determine mortality were made one and 30 min into the recovery period. Animals were considered dead if they would not right themselves if placed on their back and were not responsive to stimuli. No animal was used more than once. The carapace length of animals used ranged from 30 to 70 mm (median 45 mm) with no significant difference between treatments (Kruskal–Wallis H = 5.52, *df* = 8, *p* = 0.70).

### Data analysis

Generalised linear models (GLMs) with binomial errors were used to identify whether species or treatment were significant predictors of survival (proportion alive) at each time point (1 h, 1, 2, 4, 8, 16). To test the relative effectiveness of two different treatments at a particular time point, paired χ^2^ tests (R package: prop. test) were used to compare differences in the proportion of individuals alive. Dose response curves were plotted to illustrate changes in mortality over time and to estimate LT_50_ and LT_90_ for the treatments which did not cause 100 % mortality. All statistical analysis were carried out using ‘R’ (R Development Core Team 2012).

## Results

### *Check Clean Dry* experiment

**Fig. 1:**
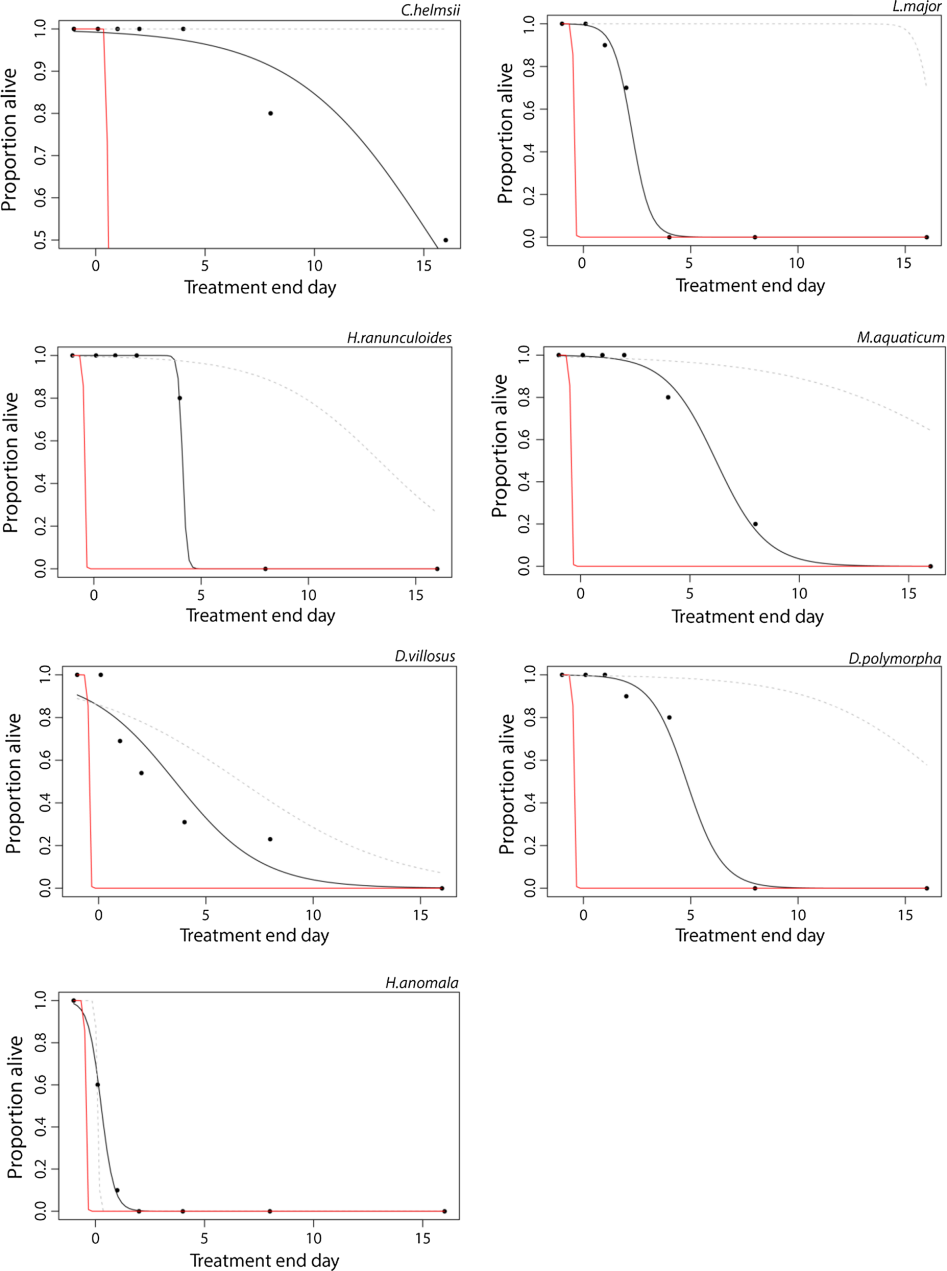
Dose response curves showing projected survival over time for hot water only (*red line*), drying (*black line* and *data points*) and control (*dashed line*) treatments. The *solid line* shows projected survival for the drying treatment. The *dashed line* shows projected survival for the control treatment and the *red line* shows projected survival for the clean treatment

Mortality differed between treatments and increased over time for all treatments. The hot water treatment and hot water and drying treatment resulted in 99 and 97 % mortality within 1 h, respectively, whereas it took 7.52 days to reach LT_90_ with the drying treatment and a projected 17.16 days to reach LT_90_ for the control group (Fig. 1, Table 2).

**Table 2:** Mean number of days taken for each species to reach 50 % mortality (LT_50_) and 90 % mortality (LT_90_) in the control and drying treatments

Species	LT 50 (days)		LT 90 (days)	
	Drying treatment	Control	Drying treatment	Control
*C.helmsii*	15.42	>100^a^	22.53	>100^a^
*H.ranunculoides*	4.13	13.35	4.34	19.04
*L.major*	2.25	16.31	3.21	17.14
*M.aquaticum*	6.19	18.52	8.73	27.65
*H.anomala*	0.15	0.10	0.95	0.10
*D.polymorpha*	4.81	16.93	6.62	23.46
*D.villosus*	3.43	6.45	8.54	15.59
MEAN	6.93	11.94^a^	7.52	17.16^a^

Results were calculated from dose–response curves

^a^As none of the *C. helmsii* died during in the control experiment, it was not possible to accurately calculate its projected survival under the control treatment. The species from was therefore excluded the mean calculation and t tests

More specifically, the hot water treatment resulted in 100 % mortality in six of the seven species and 90 % mortality in the seventh species (New Zealand pigmyweed) within 1 h, regardless of whether the nets were exposed to the air (hot water and dry treatment) or immediately put into a bag (hot water only treatment) afterwards. The hot water and dry treatment showed similar results, with 100 % mortality across 6 of the 7 species and 80 % mortality in New Zealand pigmyweed after 1 h. A much longer time period was required for the drying treatment to cause mortality, with 19 % of individuals subjected to the drying treatment still alive after 8 days and 10 % still alive after 16 days. In the control group, mortality was low with 70 % of individuals alive after 7 days and 30 % still alive after the full 16 days, among all species except bloody-red mysid. Bloody-red mysid showed high mortality (100 % within 1 day across all treatments except drying (Fig. 1).

Treatment was a significant predictor of mortality after 1 h (GLM, Estimate = 1.28, SE = 0.15, Z = 8.4, *p* < 0.001), 1 day (GLM, Estimate = 2.36, SE = 0.26, Z = 9.02, *p* < 0.001), 8 days (Estimate = 0.698, SE = 0.14, Z = 4.75, *p* < 0.001), and 16 days (Estimate = 0.624, SE = 0.17, Z = 3.59, *p* < 0.001), (Fig. 1). Species was not a significant predictor of mortality at any of the four time points (binomial GLM, *p* > 0.05).

There were no significant differences in survivorship between the hot water and dry treatment and hot water only treatment at any of the time points, indicating that drying equipment after submersion in hot water has no additional benefit (Table 3). The hot water only treatment killed a significantly higher proportion of individuals than the drying treatment or control at every time point (Table 3, Fig. 1).

**Table 3:** Results of paired X^2^ tests to compare the level of mortality (proportion) between treatments after 1 h, 1, 8 and 16 days

Treatment comparison	1 h	1 day	8 days	16 days
Clean (hot water) only versus clean (hot water) and dry	NA	2.31	NA	NA
Clean (hot water) only versus dry only	117.24***	95.68***	12.16***	7.58**
Clean (hot water) only versus control	113.77***	101.37***	70.77***	43.44***
Dry only versus control	NA	0.05	34.34***	25.20***
Clean (hot water) and dry versus control	110.03***	86.96***	70.77***	43.44***

Figures show χ^2^ value

NA = result was the same for both treatments so χ^2^ tests could not be performed

* *p* < 0.05, ** *p* < 0.01, *** *p* < 0.001

Although hot water is clearly the most effective treatment, it may not always be available to recreational water users. Drying, despite not being as effective at causing mortality as hot water (Fig. 1), caused significantly higher mortality than the control from day 4 (χ^2^ = 8.49, *p* < 0.01) onwards (Table 3), at which point the nets had dried out completely (Supplementary material Figure S1) Over half of species exposed to the drying treatment reached LT90 in 1 week (7.52 days), while aquatic plants such as curly water thyme and floating pennywort survived only 3–4 days under the drying treatment (Table 2). In contrast, New Zealand pigmyweed could survive over 23 days of drying (Table 2). Overall, drying took significantly less time to cause 50 % mortality (Independent samples T test: t = −2.76, *df* = 10, *p* < 0.05) and 90 % mortality (t = −2.89, *df* = 10, *p* < 0.05) compared to the control treatment.

### Crayfish pilot experiment

No mortalities were observed when signal crayfish were exposed to either 50 °C or 60 °C for 5 s (Table 4). With exposure to 50 °C for 5 s chronic behaviour was observed with animals inactive and unable to right themselves, however, all animals appeared to recover fully after 30 min. With exposure to 5 s at 60 °C, chronic effects were also observed and behaviour deteriorated during the recovery period.

**Table 4:** Results of the percentage mortalities observed in the heat exposure experiment with crayfish

Exposure	5 min (n = 20)		1 min (n = 15)		5 s (n = 10)	
Recovery	1 m	30 m	1 m	30 m	1 m	30 m
60 °C	100	100	65	100	0	0
50 °C	100	100	75	75	0	0
40 °C	100	100	0	0		
30 °C	0	0				

Figures expressed as percentage of crayfish in each treatment group. Recovery was measured 1 and 30 min after treatment ended for each temperature

With 1 min of exposure, mortalities were observed at 60 °C (30 min after exposure). With 75 % mortalities observed at 50 °C, but recovery being observed during the recovery period when exposed to 40 °C.

With 5 min of exposure, mortalities where observed in all animals exposed to 60, 50 and 40 °C and recovery observed at 30 °C post exposure.

## Discussion

Hot water (45 °C) caused 99 % mortality across the seven aquatic INNS used in the primary experiment within 1 h of treatment. These results demonstrate that submerging water sports equipment in 45 °C water for 15 min is an extremely effective method for killing a range of invasive non-native animals and plants in a short time frame. Moreover, hot water was effective regardless of whether or not the net which the invader was in was subsequently dried, or remained damp. New Zealand pigmyweed and parrot’s feather, were the only two species to survive submersion in hot water after 1 h, although all individuals were dead 1 day after treatment. Particular caution should be taken when using recreational equipment in areas where these plants are known to be present.

The results of our hot water experiment were similar to those of previous studies which reported 100 % mortality in zebra mussels and quagga mussels (*D. rostriformis bugensis*) in 5 min at 43 °C; adult spiny waterfleas (*B. longimanus*) in 10 min at 43 °C (Beyer et al. 2010); and spiny water flea eggs at 50 °C for ≥10 min (Branstrator et al. 2013). Although some of these INNS reached 100 % mortality in cooler temperatures or a shorter time period, we believe it is important to recommend a consistent treatment which is effective against a wide range of species, without the need for waterusers to know which INNS are present. As 45 °C for 15 min was identified as the most efficient time/temperature combination to cause 100 % mortality in killer shrimp (Stebbing et al. 2011), we recommend that this longer time period is used as a consistent treatment.

Adult crayfish are unlikely to remain attached to equipment without being noticed, but were used in this study as a proxy for juvenile crayfish. Although 100 % mortalities were observed when crayfish were exposed to 60 °C for 1 min, this water temperature could degrade watersports equipment and has the potential to cause burns in children (Feldman et al. 1998). With 100 % mortality observed with 5 min exposure at 40 °C, the recommendation of exposing water sport equipment in 45 °C water for 15 min is considered more than sufficient to cause mortality in juvenile crayfish.

In the absence of hot water, drying was still found to be a significantly more effective treatment than doing nothing (control) and caused 90 % mortality in a mean of 7.52 days in all species except New Zealand pigmyweed, suggesting that it would be suitable as a biosecurity treatment for anglers who go fishing once a fortnight or less frequently in areas where New Zealand pigmyweed is not present. Our desiccation treatment took longer to cause mortality in plants than previous studies. For example, drying Eurasian water milfoil (*Myriophyllum spicatum*), resulted in 71 % mortality within 1 h and 100 % mortality within 1 day (Jerde et al. 2012). In an animal experiment, air exposure of ≥6 h prevented the dormant egg stages of spiny waterflea from hatching (Branstrator et al. 2013). In contrast, the plants in our study took at least of 2.25 days to reach LT_50_ and 3.21 to reach LT_9_ and the animals took at least 22 h to reach LT_90_ (Table 2). The longer time-to-mortality in our desiccation treatment is likely to be due to the plant fragments and animals remaining enclosed in damp nets which retained water for a number of days after initial submersion (Figure S1, supplementary material) whereas the plants/animals were not enclosed in the aforementioned studies. Our results demonstrate that drying can take many days, particularly for INNS entrapped in large equipment and in cool or damp conditions and is a more subjective biosecurity treatment (i.e. people’s perceptions of what ‘dry’ is may vary). These results support previous studies which show that complete desiccation is required for it to be effective (Jerde et al. 2012; Poznanska et al. 2013), making it an unsuitable decontamination method for use by anglers who go fishing frequently.

Despite some mortality, six of the seven species (all except bloody-red mysid) in the control group were able to survive for at least 16 days in damp conditions. As recent research suggests that 64 % of anglers visit multiple catchments within a fortnight (Anderson et al. 2014), this demonstrates the potential for invaders to survive in damp equipment in the absence of biosecurity. Several of the species in this experiment were not previously thought to be able to survive for this long out of water: killer shrimp has only been reported to survive for 15 days out of water (Fielding 2011) and zebra mussels for 3–5 days (Ricciardi et al. 1995). Our results also demonstrate that aquatic plants including floating pennywort and parrot’s feather can survive out of water for at least 16 days which, to the best of our knowledge, has not been previously reported. Unlike the other species tested, bloody-red mysid showed high mortality (100 % within 1 day in all treatments except drying). This species appeared particularly fragile, so it is probable that handling in the laboratory or physical damage by the nets resulted in mortality. Based on our results, it seems unlikely that bloody-red mysid would survive transport in an angling net, therefore water-based transfer methods (such as the ballast water of boats) may be more important vectors for this species; as presumed for its introduction into the Great Lakes (Brooking et al. 2010).

Hot water provides a rapid and easy method to clean equipment as part of the *Check Clean Dry* protocol and we believe it is a simple and effective method to recommend to the anglers (e.g. 78 % of those in the UK) who do not currently clean their kit after use (Anderson et al. 2014). While we have demonstrated the effectiveness of this method at killing a range of INNS, we stress that further research must be conducted to test the effectiveness of hot water as a treatment to kill aquatic pathogens, such as *Gyrodactlylus salaris,* a salmon ectoparasite which is considered to be the most important exotic fish-disease threat to the UK (Peeler et al. 2004); *Aphanomyces astaci*, the causal agent of crayfish plague (Oidtmann et al. 2002) and *Batrachochytrium dendrobatidis,* the causal agent of chytrid disease in amphibians (Johnson and Speare 2003). We acknowledge that aquatic parasites such as these pose a similar economic and ecological risk to INNS and that anglers using equipment in multiple countries pose a risk of parasite dispersal (Anderson et al. 2014). We advocate the continued use of Virkon Aquatic^®^ (DuPont 2014) as a biosecurity agent for anglers travelling between countries or using equipment in areas where aquatic parasites may be present. Further work into the effectiveness of hot water as a control measure for parasites would be of significant use in demonstrating hot water as a single ‘catch all’ biosecurity message for both invasive species and aquatic pathogens.

## Conclusion

Hot water fulfils the criteria for an effective biosecurity treatment. Not only does it cause 99 % mortality within an hour, it is environmentally sound and cost effective (O’Neill and MacNeill 1991; Beyer et al. 2010; Stebbing et al. 2011; Perepelizin and Boltovskoy 2011) and the recommended temperature of 45 °C, is below the temperature at which hot water is thought to be able to cause burns in children (52 °C) making it safe to use by children as well as adults (Feldman et al. 1998). However, we recommend that water is disposed of on land and away from a water source.

These results provided evidence that hot water is effective at killing a range of high impact invasive species in a short time frame. The use of hot water (45 °C for 15 min) for the ‘Clean’ stage of the UKs national *Check Clean Dry* biosecurity awareness campaign would greatly enhance biosecurity efforts. In addition to anglers, this method could be used by water sports participants with wetsuits or equipment that can easily be submerged, as well as ecologists, environmental scientists and field centre staff and volunteers who use nets, waders and other equipment to undertake freshwater fieldwork in the UK.

## Electronic supplementary material

Below is the link to the electronic supplementary material.

Supplementary material 1 (DOCX 42 kb)
