# Supplementary material for: Invaders in hot water: a simple decontamination method to prevent the accidental spread of aquatic invasive non-native species
Source: Biol Invasions. 2015 Mar 26;17(8):2287–97. doi: 10.1007/s10530-015-0875-6 (PMC4544425; doi:10.1007/s10530-015-0875-6)
Supplement: Supplementary file 1 — Supplementary material 1 (DOCX 42 kb) [file 10530_2015_875_MOESM1_ESM.docx]

**­Supplementary Material**

|  |
| --- |
| **Figure S1**. The graph shows the mean weight loss measured in a subset of twenty randomly selected nets from each treatment at each time point. Error bars show standard error around the mean. CL=hot water only treatment; CLDRY=hot water treatment followed by drying treatment; DRY = drying only treatment; CON=control group. |

ANOVA tests were performed in R version 2.15 (R Development Core Team 2012) to determine whether the differences in weight in the nets exposed to drying treatments or non-drying treatments were significantly different. At each time point, 80 bags (40 from drying treatments and 40 from non-drying treatments) were due to end at that time point were randomly selected and weighed. From day 1 onwards, there was a significant difference in the weight of the bags exposed to drying vs. bags which were prevented from drying (Table S1).

| **Table S1.** Results of ANOVA tests to compare the weights of nets which were subjected to drying treatments (hot water and dry treatment; dry only treatment) and the nets which were prevented from drying by being stored in unsealed plastic bags (hot water treatment; control). N = 80 nets at each time point. | |
| --- | --- |
| Time point | Results of ANOVA test |
| 1h | F_1,78_ = 11.79, p<0.01 |
| 1 day | F_1,78_ = 46.95, p<0.0001 |
| 2 days | F_1,78_ = 50.25, p<0.0001 |
| 4 days | F_1,78_ = 61.43, p<0.0001 |
| 8 days | F_1,78_ = 79.04, p<0.0001 |
| 16 days | F_1,78_ =130.9, P<0.0001 |

Reference: R Development Core Team (2012) R: A Language and Environment for Statistical Computing. R Foundation for Statistical Computing, Vienna, Austria
